# Indian Essential Medicine List for Children: Time to Revisit

**DOI:** 10.7759/cureus.35340

**Published:** 2023-02-22

**Authors:** Shubha Singhal, Siddhartha Dutta, Zalak Thakker, Rima Shah

**Affiliations:** 1 Pharmacology, All India Institute of Medical Sciences, Rajkot, IND; 2 Attending Pediatrician, Bassett Healthcare Network, Cooperstown, USA

**Keywords:** out of pocket expenditure, rational use of medicine, pediatric, availability, aware

## Abstract

Background: The majority of the under five mortality rate (U5MR) in India were due to treatable causes and could have been prevented by providing quality medicines. Availability and affordability of medicine can be improved by the introduction of essential medicine concepts.

Purpose: The current study was carried out to compare the latest edition of the WHO essential medicine list for children (EMLc) with that of Indian EMLc to determine the need to update the Indian EMLc.

Methods: A descriptive observational study was carried out in the Department of Pharmacology of a tertiary care hospital. The latest edition of WHO EMLc (8^th^) was compared with the latest edition of Indian EMLc (1^st^) in terms of inclusion of categories or subcategories, the number of medicines in each category or subcategories, medicines which are present in WHO EMLc but missing in Indian EMLc and vice versa.

Results: In total 134 medicines are present in Indian EMLc as compared to 350 medicines in WHO EMLc. The important categories which are completely missing in Indian EMLc are medicines for reproductive health and perinatal care, peritoneal dialysis solution, medicines for mental and behavioral disorders, and medicines for diseases of joints. The important medicines which are not included in Indian EMLc are bedaquilline, delaminid, cefixime, piperacillin+tazobactum, vancomycin, acyclovir, azathioprine, cisplatin, and filgrastim. Important vaccines including rotavirus, cholera, hepatitis, and typhoid vaccine are not mentioned in Indian EMLc.

Conclusion: There is an urgent need to update the Indian EMLc in order to promote access to pediatric medicine and facilitate the rational use of medicines.

## Introduction

India was among five countries in the world that contributed to nearly half of all under-five deaths that occurred in 2019. The majority of these deaths were due to treatable conditions and could have been prevented by providing access to quality medicines and affordable care [1]. India has declared the “right to health” as a basic right of Indian citizens. "Right to health" means that every person has the right to achieve the best possible level of health [2]. The goal of India's National Health Policy, 2021 is to achieve the highest possible level of health and well-being for all people of all ages by integrating preventive and promotive healthcare into all developmental policies and providing universal access to high-quality healthcare services to citizens without financial hardship [3].

As per Census 2011, 28.5% population of India lies between the 0 and 14 age group by the year 2021, making pediatric population a significant part of the community in India [3]. A survey conducted in India reported that only 53% of the pediatric medicines/formulations were available in the public health facility of India [4]. In the absence of medicines for the pediatric population in public health facilities, caregivers are either forced to opt for the private sector treatment which is costly leading to out-of-pocket expenditure (OOPE) or forego the treatment at all; contributing to pediatric morbidity and mortality in India. OOPE is the main source of funding even though the public opts for the health insurance schemes and spending on medicines accounts for about 72% of OOPE and lends a significant financial burden leading to increased poverty [5-6].

The cost of treatment has risen in India, resulting in inequalities in access to healthcare services. India spends barely 1.28% of its GDP on health in the 2017-2018 financial year. In nominal terms, per capita, public health expenditure increased from Rs 621 in 2009-2010 to Rs 1657 in 2017-2018 [3]. In India, healthcare-related financial burdens cause around one-third of individuals to fall into poverty each year, which is concerning given that nearly two-thirds of the population is already impoverished or living in poverty [6-7]. Given the dependency of pediatric patients on their parents/caretakers for their health needs, treatment with high costs has a huge impact on the financial situation. Hence it is crucial that pediatric medicines are made available at affordable prices in society.

One intervention that can help to improve the availability and affordability of medicine is the introduction of the essential medicine concept in the healthcare sector. Essential medicines are those medicines that satisfy the priority healthcare needs of the population and are intended to be available in all healthcare systems at all times in adequate amounts, in the appropriate dosage forms, with assured quality, and at a price, the individual and the community can afford [6]. To improve the availability and affordability of medicine in children WHO introduced the essential medicine list for children (EMLc) in 2007, 30 years after the introduction of the adult essential medicine list. The purpose of EMLc is to ensure that medicines in the health system are prioritized in an evidence-based manner to meet children's needs [7]. The WHO Model Lists of Essential Medicines are updated every two years by the Expert Committee on Selection and Use of Essential Medicines. Currently, 8th EMLc is in practice which was introduced in the year 2021 [8]. Indian Academy of Paediatrics (IAP) has also introduced Indian EMLc in October 2011 in collaboration with WHO. The IAP's EMLc of India meets the fundamental needs of the vast majority of children seeking medical care in India. The drugs were chosen based on WHO selection criteria for essential medicines and Indian National Health Programs. It consists of 134 medicines. At the time of introduction, it was proposed to revise the list regularly for at least every two years. However, to date, it is not revised at all [9]. The purpose of this study is to compare the list of WHO EMLc with IAP EMLc and to find out the need to update the Indian EMLc to promote access to pediatric medicine.

## Materials and methods

This was an observational and descriptive study carried out in the Department of Pharmacology of a tertiary care teaching hospital from February 2022 to May 2022. The study compared the latest edition of Indian EMLc (1st) with WHO EMLc (8th) to find out the need for an update of Indian EMLc. As it was an observational study comparing the two public databases, hence ethics committee permission was not required.

The latest edition of WHO EMLc (8th list) is published by World Health Organization in 2021 and consists of medicines that are most effective and safe in children up to 12 years of age and meet society’s unmet needs. There are two parts to this list: the core list and the complementary list. The core list includes medicines that are the most cost-effective for a specific health issue and that can be administered with a minimal amount of medical resources. The complementary list consists of medicines that require specialized diagnostic or monitoring facilities or trained healthcare providers. In addition, it also consists of medicines which have a lower cost-benefit ratio.

The WHO EMLc consists of various categories and subcategories. Each category and subcategory include medicines that are considered to be the most efficient and safe for use in children up to the age of 12 years. In front of each medicine, the strength and dosage form which is appropriate for children is mentioned. The list also consists of therapeutic alternative for various medicines [8].

The WHO EMLc acts as a model list and countries can make their own EMLc by taking a core concept from WHO EMLc. IAP also made Indian EMLc in 2011. The IAP's EMLc of India meets the fundamental needs of the vast majority of children seeking medical care in India. The drugs were chosen based on WHO selection criteria for essential medicines and Indian National Health Programs. It consists of 134 medicines [9].

For the present study, the WHO EMLc 2021 (8th list) was employed as a standard reference list for comparison, whereas the Indian EMLc 2011 (1st list) was used as the comparator list. Due to the fact that neither of the lists was completely super imposable, a spreadsheet was developed to facilitate head-to-head comparison. Both the lists were compared for the following parameters:

1) Presence of category or subcategories

2) Number of medicines in each category or sub-category

3) Medicines which are present in WHO EMLc but missing in Indian EMLc

4) Medicines which are present in Indian EMLc but missing in WHO EMLc

If a medicine was not mentioned in one therapeutic section but was available in a different therapeutic section, it was noted as available.

## Results

**Figure 1:**
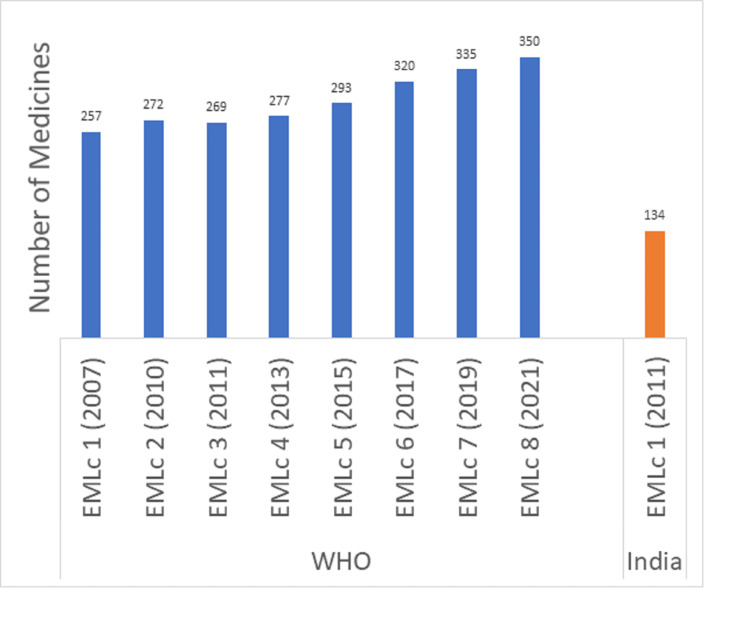
Number of updates of WHO EMLc and Indian EMLc. EMLc, essential medicine list for children

A comparative study was carried out to determine the necessity for an updating of the Indian EMLc. The first WHO EMLc was published in 2007 and has been amended seven times since then (Figure 1).

The 8th WHO EMLc is currently in use, and it contains 350 medications, including 24 vaccines. The WHO EMLc 2021 includes the AWaRe (Access, Watch, Reserve) classification of antibiotics, which was developed by WHO in 2019 to address antibiotic resistance. The WHO EMLc also suggests therapeutic alternatives for specific drugs that are similar in clinical effectiveness and safety to the primary drug (Table 1).

**Table 1:** Major difference between the 8th WHO EMLc and 1st Indian EMLc. EMLc, essential medicine list for children; AWaRe, Access, Watch, Reserve

Parameters	WHO EMLc	Indian EMLc
Origin	2007	2011
Number of revision	Seven times	Nil
Current list	Eighth	First
Total number of medicines	350	134
Number of vaccines	24	7
Therapeutic alternatives	Given	Not given
AWaRe classification of antibiotics	Yes	No

Indian EMLc was released in 2011 and has not been revised at all. It neither included AWaRe classification of antibiotics nor therapeutics alternatives (Table 1). 

In total 29 categories are present in WHO EMLc 2021 while Indian EMLc has 22 categories. The categories which are completely missing in Indian EMLc are blood products of human origin and plasma substitutes, diagnostic agents, medicines for reproductive health and perinatal care, peritoneal dialysis solution, medicines for mental and behavioral disorder, medicines for diseases of joints, and dental preparation (Table 2).

**Table 2:** Comparison of categories, subcategories, and number of medicines among 8th WHO EMLc and 1st India EMLc. *No separate subcategory. Medicinal gas includes under the category of inhalational medicines. + present; -- absent EMLc, essential medicine list for children; NSAIMs, non-opioids and non-steroidal anti-inflammatory medicines; DMARDs, disease modifying anti-rheumatic drugs

Categories	WHO EMLc	India EMLc	Number of medicines in WHO EMLc	Number of medicines in India EMLc
Anesthetics, preoperative medicines, and medical gases	+	+	12	8
Inhalational medicines	+	+	4	3
Injectable medicines	+	+	2	1
Local anesthetics	+	+	3	2
Preoperative medication and sedation for short-term procedures	+	+	3	2
Medical gases*	+	--	1	0
Medicines for pain and palliative care	+	+	15	3
NSAIMs	+	+	2	2
Opioid analgesic	+	+	2	1
Medicines for other symptoms common in palliative care	+	--	11	--
Antiallergics and medicines used in anaphylaxis	+	+	5	5
Antidotes and other substances used in poisonings	+	+	10	4
Nonspecific	+	+	1	1
Specific	+	+	9	3
Anticonvulsants/Antiepileptics	+	+	9	7
Anti-infective medicines	+	+	126	49
Intestinal anthelminthics	+	+	7	2
Antifilariasis	+	+	3	1
Antischistosomals and other antitrematode medicines	+	--	3	--
Cysticidal medicines	+	--	3	--
Antibacterials (except anti-leprosy and anti-tuberculosis)	+	+	35	13
Antileprosy medicine	+	+	3	3
Anti-tuberculosis medicines	+	+	21	5
Antifungal medicines	+	+	9	1
Antiherpes medicines	+	--	1	--
Antiretrovirals	+	+	14	13
Medicines for Hepatitis B	+	--	1	--
Medicines for Hepatitis C	+	--	5	--
Antiamoebic and antigiardiasis medicines	+	+	2	2
Antileishmaniasis medicines	+	+	4	3
Antimalarial medicine	+	+	18	6
Antipneumocystosis and antitoxoplasmosis	+	--	3	--
African trypanosomiasis	+	--	6	--
American trypanosomiasis	+	--	2	--
Medicines for ectoparasitic infections	+	--	1	--
Antimigraine medicine	+	+	3	3
Immunomodulators and antineoplastics	+	+	46	7
Immunomodulators for non-malignant disease	+	--	4	--
Antineoplastics	+	+	42	7
Medicines affecting the blood	+	+	12	2
Antianemia medicines	+	+	4	2
Medicines affecting coagulation	+	--	6	--
Other medicines for hemoglobinopathies	+	--	2	--
Blood products of human origin and plasma substitutes	+	--	10	--
Blood and blood component	+	--	4	--
Human immunoglobulins	+	--	3	--
Blood coagulation factors	+	--	2	--
Plasma substitutes	+	--	1	--
Cardiovascular medicines	+	+	4	4
Antihypertensive medicines	+	--	1	--
Medicines used in heart failure	+	+	3	4
Dermatological medicines (topical)	+	+	16	6
Antifungal medicine	+	+	2	1
Anti-infective medicine	+	+	3	1
Anti-inflammatory and antipruritic medicines	+	+	3	2
Medicines affecting skin differentiation and proliferation	+	--	6	--
Scabies and pediculicides	+	+	2	2
Diagnostic agents	+	--	3	--
Ophthalmic medicines	+	--	2	--
Radiocontrast media	+	--	1	--
Antiseptics and disinfectants	+	+	7	2
Antiseptics	+	+	3	1
Disinfectants	+	+	4	1
Diuretics	+	+	4	3
Gastrointestinal medicines	+	+	9	4
Anti-ulcer medicine	+	+	2	1
Antiemetic medicines	+	+	4	1
Medicine used in diarrhea	+	+	3	2
Medicines for endocrine disorders	+	+	13	4
Adrenal hormones and synthetic substitutes	+	+	2	2
Medicines for diabetes	+	+	4	2
Medicines for hypoglycemia	+	--	2	--
Thyroid hormones and antithyroid medicines	+	+	5	1
Immunologicals	+	+	29	10
Diagnostic agents	+	--	1	--
Sera, immunoglobulins, and monoclonal antibodies	+	+	4	3
Vaccines	+	+	24	27
Muscle relaxants (peripheral acting) AND cholinesterase inhibitors	+	+	4	2
Ophthalmological preparations	+	+	11	3
Anti-infective agents	+	+	7	1
Anti-inflammatory agents	+	+	1	1
Local anesthetics	+	--	1	--
Mydriatics	+	+	2	1
Medicines for reproductive health and perinatal care	+	--	5	--
Medicines administered to the neonate	+	--	5	--
Peritoneal dialysis solution	+	--	1	--
Medicines for mental and behavioral disorders	+	--	3	--
Medicines used in psychotic disorders	+	--	2	--
Medicines used in mood disorders	+	--	2	--
Medicines acting on respiratory tract	+	+	3	2
Anti-asthmatic medicines	+	+	3	2
Solutions correcting water, electrolyte, and acid base disturbances	+	+	9	8
Oral	+	+	2	1
Parenteral	+	+	6	6
Miscellaneous	+	+	1	1
Vitamins and minerals	+	+	9	4
Ear, nose and throat medicines	+	+	4	3
Medicines for diseases of joints	+	--	3	--
DMARDs	+	--	2	--
Juvenile joint disease	+	--	1	--
Dental preparations	+	--	3	--

In addition, important subcategories including medicine for common symptoms in palliative care, immunomodulators, medicines for hepatitis B, medicines for hepatitis C, medicines affecting coagulation, medicines affecting skin differentiation and proliferation and anti-hypertensive medicines are absent in Indian EMLc (Table 2).

The number of medicines are far less in Indian EMLc as compared to WHO EMLc. Indian EMLc includes 49 anti-infective medicines as compared to 129 medicines of WHO EMLc. Similarly, Indian EMLc includes only three medicines in the category of medicines for pain and palliative care while WHO EMLc includes 15 medicines in the same category. The other categories which have major differences in terms of number of medicines are immunomodulators and antineoplastics, medicines affecting blood, medicines for endocrine disorder, immunologicals, and ophthalmological preparations (Table 2).

There are medicines which are present in WHO EMLc but absent in Indian EMLc. Major anti-infective medicines which are not included in Indian EMLc are bedaquilline, delaminid, cefixime, cefotaxime, piperacillin+tazobactum, vancomycin, acyclovir, oseltamivir, etc. (Table 3). Ciclosporin, azathioprine, cisplatin, paclitaxel, filgrastim are few examples of anti-neoplastic drugs which are missing from Indian EMLc. Important vaccines including rotavirus vaccine, cholera vaccine, hepatitis vaccine, meningococcal meningitis vaccine, typhoid vaccine are also not mentioned in Indian EMLc. There are 19 medicines which are present in Indian EMLc but absent in WHO EMLc (Table 3).

**Table 3:** Comparison of presence or absence of medicines in WHO EMLc and India EMLc. *Medicines which are not present in that category, but present in some other category EMLc, essential medicine list for children; HPV, human papilloma virus; ATRA, all-trans retinoid acid

Categories	Medicines which are not included in India EMLc but present in WHO EMLc	Medicines which are not included in WHO EMLc but present in India EMLc
Anesthetics, preoperative medicines, and medical gases	Isoflurane*	
	Propofol	
	Bupivacaine	
	Morphine	
Medicines for pain and palliative care	Methadone	
	Amitriptyline	
	Cyclizine	
	Dexamethasone*	
	Diazepam*	
	Docusate sodium	
	Fluoxetine	
	Hyoscine hydrobromide	
	Lactulose	
	Midazolam*	
	Ondansetron	
	Senna	
Antiallergics and medicines used in anaphylaxis	Loratadine	Chlorphenamine
Antidotes and other substances used in poisonings	Acetylcysteine	Pralidoxime
	Calcium gluconate	
	Deferoxamine	
	Dimercaprol	
	Fomepizole	
	Sodium calcium edetate	
	Succimer	
Anticonvulsants/Antiepileptics	Lamotrigine	
	Ethosuximide	
Anti-infective medicines		
Intestinal anthelminthics	Ivermectin	
	Levamisole	
	Mebendazole	
	Niclosamide	
	Praziquantel	
	Antifilariasis	Albendazole*	
Ivermectin		
Antischistosomals and other antitrematode medicines	Praziquantel	
	Triclabendazole	
	Oxamniquine	
Cysticidal medicines	Albendazole*	
	Mebendazole	
	Praziquantel	
Antibacterials (except anti-leprosy and anti-tuberculosis)	Amikacin	
	Cefalexin	
	Cefazolin	
	Chloramphenicol	
	Clindamycin	
	Cloxacillin	
	Doxycycline*	
	Nitrofurantoin	
	Phenoxymethylpenicillin	
	Trimethoprim	
	Cefixime	
	Cefotaxime	
	Cefuroxime	
	Clarithromycin	
	Piperacillin + Tazobactam	
	Vancomycin	
	Ceftazidime	
	Meropenem	
	Ceftazidime + Avibactam	
	Colistin	
	Fosfomycin	
	Linezolid	
	Polymyxin B	
Antileprosy medicine		
Anti-tuberculosis medicines	Isoniazid + pyrazinamide + rifampicin	
	Isoniazid + rifampicin	
	Isoniazid + rifapentine	
	Rifapentine	
	Amikacin	
	Amoxicillin + clavulanic acid*	
	Bedaquiline	
	Cycloserine	
	Delaminid	
	Ethionamide	
	Levofloxacin	
	Linezolid	
	Meropenem	
	Moxifloxacin	
	p-amino salicylic acid	
Antifungal medicines	Amphotericin B*	
	Flucytosine	
	Griseofulvin	
	Itraconazole	
	Nystatin	
	Voriconazole	
	Micafungin	
	Potassium Iodide	
Antiherpes medicines	Acyclovir	
Antiretrovirals	Darunavir	Abacavir
	Dolutegravir	Didanosine
	Raltegravir	Efavirenz
	Abacavir+ lamivudine	Atazanavir
	Isoniazid +pyridoxine +sulfamethoxazole+ trimethoprim	Stavudine+lamivudine
	Ribavirin	Stavudine+lamivudine+nevirapine
	Oseltamivir	Zidovudine +lamivudine+nevirapine
	Valganciclovir	Abacavir
Medicines foe Hepatitis B	Entecavir	
Medicines for Hepatitis C	Daclatasvir	
	Daclatasvir+Sofosbuvir	
	Glecaprevir+pibrentasvir	
	Sofosbuvir	
	Sofosbivir+velpatasvir	
Antiamoebic and antigiardiasis medicines		
Antileishmaniasis medicines	Miltefosine	Pentamidine isethionate
	Paromomycin	
Antimalarial medicines	Amodiaquine	Artesunate+sulfadoxine +Pyrimethamine
	Artemether	
	Artesunate	
	Artesunate+ amodiaquine	
	Artesunate+ mefloquine	
	Artesunate + pyronaridine tetraphosphate	
	Dihydroartemisinin + piperaquine phosphate	
	Mefloquine	
	Sulfadoxine + pyrimethamine	
Antipneumocystosis and antitoxoplasmosis	Pyrimethamine	
	Sulfadiazine	
	Sulfamethoxazole + trimethoprim	
African trypanosomiasis	Fexinidazole	
	Pentamidine	
	Suramin Sodium	
	Eflornithine	
	Nifurtimox	
	Melarsoprol	
American trypanosomiasis	Benznidazole	
	Nifurtomox	
Medicines for ectoparasitic infections	Ivermectin	
Antimigraine medicine	---	--
Immunomodulators and antineoplastics	Adalimumab	
	Azathioprine	
	Ciclosporin	
	Tacrolimus	
	Arsenic trioxide	
	Asparaginase	
	Bleomycin	
	Calcium folinate	
	Carboplatin	
	Cisplatin	
	Cytarabine	
	Dacarbazine	
	Dactinomycin	
	Doxorubicin	
	Etoposide	
	Fluorouracil	
	Hydroxycarbamide	
	Ifosfamide	
	Irinotecan	
	Oxaliplatin	
	Paclitaxel	
	Pegaspargase	
	Procarbazine	
	Realgar- Indigo naturalis formulation	
	Tioguanine	
	Vinblastine	
	Vinorelbine	
	ATRA	
	Dasatinib	
	Everolimus	
	Imatinib	
	Nilotinib	
	Rituximab	
	Filgrastim	
	Dexamethasone*	
	Hydrocortisone*	
	Allopurinol	
	Mesna	
	Rasburicase	
	Adalimumab	
	Azathioprine	
	Ciclosporin	
	Tacrolimus	
	Arsenic trioxide	
	Asparaginase	
	Bleomycin	
	Calcium folinate	
	Carboplatin	
	Cisplatin	
	Cytarabine	
	Dacarbazine	
	Dactinomycin	
	Doxorubicin	
	Etoposide	
	Fluorouracil	
	Hydroxycarbamide	
	Ifosfamide	
	Irinotecan	
	Oxaliplatin	
	Paclitaxel	
	Pegaspargase	
	Procarbazine	
	Realgar-Indigo naturalis formulation	
	Tioguanine	
	Vinblastine	
	Vinorelbine	
	ATRA	
	Dasatinib	
	Everolimus	
	Imatinib	
	Nilotinib	
	Rituximab	
	Filgrastim	
	Dexamethasone*	
	Hydrocortisone*	
	Allopurinol	
	Mesna	
	Rasburicase	
Medicines affecting the blood	Ferrous salt	
	Folic acid	
	Hydroxocobalamin	
	Erythropoiesis stimulating agents	
	Enoxaparin	
	Phytomenadione	
	Desmopressin	
	Heparin sodium	
	Protamine sulfate	
	Warfarin	
	Deferoxamine	
	Hydroxycarbamide	
Blood products of human origin and plasma substitutes	Fresh frozen plasma	
	Platelets	
	Red blood cells	
	Whole blood	
	Anti-rabies immunoglobulin*	
	Anti-tetanus immunoglobulin*	
	Normal immunoglobulin	
	Coagulation factor VIII	
	Coagulation factor IX	
	Dextran 70	
Cardiovascular medicines	Enalapril	Spironolactone*
Dermatological medicines (topical)	Terbinafine	
	Mupirocin	
	Potassium permanganate	
	Betamethasone	
	Benzoyl peroxide	
	Calcipotriol	
	Coal tar	
	Podophyllum resin	
	Salicylic acid	
	Urea	
Diagnostic agents	Fluorescein	
	Tropicamide	
	Barium sulfate	
Antiseptics and disinfectants	Chlorhexidine	
	Ethanol	
	Alcohol based hand rub	
	Chlorine based compound	
	Glutaral	
Diuretics	Hydrochlorothiazide	
Gastrointestinal medicines	Ranitidine	
	Dexamethasone*	
	Ondansetron	
	Aprepitant	
	Oral rehydration salt-zinc sulfate	
17) Medicines for endocrine disorders	Fludrocortisone	
	Long acting insulin analogues	
	Metformin	
	Diazoxide	
	Glucagon	
	Lugol’s solution	
	Methimazole	
	Potassium iodide	
	Propylthiouracil	
Immunologicals	Tuberculin, purified protein derivative	
	Diphtheria	
	Diphtheria vaccine (available as DPT)	
	Hemophilus influenzae type b vaccine	
	HPV vaccine	
	Pertusis vaccine (available as DPT)	
	Pneumococcal vaccine	
	Rotavirus vaccine	
	Rubella vaccine	
	Japanese encephalitis vaccine	
	Tick borne encephalitis vaccine	
	Yellow fever vaccine	
	Cholera vaccine	
	Hepatitis vaccine	
	Meningococcal meningitis vaccine	
	Typhoid vaccine	
	Influenza vaccine	
	Mumps vaccine	
	Varicella vaccine	
Muscle relaxants (peripheral acting) AND cholinesterase inhibitors	Vecuronium	
	Pyridostigmine	
Ophthalmological preparations	Aciclovir	
	Azithromycin*	
	Erythromycin	
	Natamycin	
	Ofloxacin	
	Tetracycline	
	Tetracaine	
	Adrenaline*	
Medicines for reproductive health and perinatal care	Caffeine citrate	
	Chlorhexidine	
	Ibuprofen*	
	Prostaglandin E1	
	Surfactant	
Peritoneal dialysis solution	Intraperitoneal dialysis solution	
Medicines for mental and behavioral disorders	Chlorpromazine	
	Haloperidol	
	Fluoxetine	
Medicines acting on respiratory tract	Epinephrine (adrenaline)*	
Solutions correcting water, electrolyte, and acid base disturbances	Potassium chloride*	Ringer lactate solution
	Sodium hydrogen carbonate	Sodium bicarbonate
	Sodium lactate, compound solution	
Vitamins and minerals	Ascorbic acid	Multivitamin
	Iodine	Vitamin K
	Multiple micronutrient powder	
	Pyridoxine	
	Riboflavin	
	Thiamine	
	Calcium gluconate	
Ear, nose and throat medicines	Acetic acid	
Medicines for diseases of joints	Hydroxychloroquine	
	Methotrexate*	
	Acetylsalicylic acid	
Dental preparations	Fluoride	
	Glass ionomer cement	
	Silver diamine fluoride	

## Discussion

The medications usually are discovered or developed considering the need or burden of a particular disease in the society. Moreover, the drugs are usually developed for the adult population and paediatric medications have always been a grey area. The present study compared the essential medicine list for children composed by WHO with the list created by IAP in India. The Indian EMLc contained lesser categories and number of medications in contrast to WHO EMLc. It is also crucial to note that important antimicrobials, anticoagulants, immunomodulators and antineoplastics, vaccines and anti-hypertensives were among many categories that were lacking in the Indian EMLc. There were also few medications, majority of which were antimicrobials/antivirals that were exclusively observed in the Indian EMLc as compared to WHO EMLc.

The current study observed that the update of WHO EMLc has been has been done from time to time but the case was not identical with Indian EMLc [8-10]. As per WHO, essential medicines for a population, meet the primacy health care requirements and are selected on the basis of prevalence and public health significance of a disease with proven efficacy and safety [11]. This EMLc is updated every two years, and serves as a guidance document for various countries to modify their own EML in accordance with local disease burden and treatment priorities. Given the fact, that Indian EMLc has not been updated since a long time, the very essence of EMLc to be updated from time to time according to the health needs takes a toll and stays far from accomplished.

The AWaRe classification of antibiotics that was formulated by WHO in 2019 to curb antibiotic resistance is also included in the WHO EMLc whereas the Indian EMLc lacks any such modifications [11-12]. In contrast to the Indian EMLc, WHO EMLc also proposes alternate therapeutic substitutes for specific classes with equivalent clinical efficacy and safety as compared to the principal drug [9].

The Indian EMLc lacked many important categories of drugs like blood products and plasma substitutes, diagnostic agents, reproductive and perinatal care, mental and behavioural disorder, rheumatic diseases and dental preparation and few subcategories like few medications for palliative care, immunomodulators, hepatitis B, hepatitis C, coagulation, skin differentiation and proliferation and anti-hypertensives when compared to the WHO list [9, 11]. A study conducted by Roy et al reported similar finding and also pointed out the missing categories of drugs from the Indian EMLc as compared to WHO EMLc [4]. These missing list of medications in the EMLc can serve as a hurdle for the prescribers in the primary health care sector who might follow the EMLc to prescribe the patients considering to also practise rational use of medication in the population.

The current study also points out that the total number of drugs are considerably lower in Indian EMLc as compared to WHO. The lesser number of drugs in the list can restrict the healthcare physicians to prescribe freely as they will be left with a limited bunch of choices. Crucial antimicrobials like bedaquilline, delaminid, cefixime, cefotaxime, piperacillin+tazobactum, vancomycin, acyclovir, oseltamivir etc, anticancers and immunomodulators like ciclosporin, azathioprine, cisplatin, paclitaxel, filgrastim, important vaccines for children like rotavirus, cholera, hepatitis, meningococcal and typhoid were also missing from the Indian EMLc. The reason of non-inclusion of few of the above listed medications and vaccines could be that they are recently approved drugs or the use of the medication or vaccines have increased in the recent past and were not that commonly needed during the formulation of the Indian EMLc which was developed long back.

As the EMLc is developed considering the local burden of diseases and hence every country can modify the EMLc and add medications as per their need as directed by WHO [12]. The Indian EMLc also had been formulated following the same concept and therefore has 19 medications which majorly includes antiretrovirals, antileishmaniasis, antimalarial, multivitamin, vitamin K, iron and folic acid, ringer lactate solution, anti-tetanus immunoglobulin etc. considering the local needs and demand of the Indian population [10].

Majority of the studies conducted in India on the essential medicines are conducted on comparing the WHO and Indian EMLc medications with the availability of these medications in the healthcare system and formularies of different hospitals round the country but none of them compared the WHO and Indian EMLc to assess the differences and generate evidence [13-15].

To our knowledge, there are limited studies conducted on the EMLc and there is lack of study comparing the Indian and WHO EMLc in the Indian scenario. This study brings out the differences, lacunes and area of improvements needed in the Indian list as compared to the WHO. This study will serve as a reference for further studies and also help the policy makers of the healthcare system and drugs in India to formulate and revise newer policies. The findings of the study will also assist the the National Pediatric Associations in India to revise and formulate an updated EMLc for the healthcare system in order to facilitate rational use of medicines and betterment of the society.

## Conclusions

Essential medicine lists are very important tool for providing quality medical care to patients by doctors, avail health care, and treatment at affordable cost by patients and helps in appropriate budget allocation for health by the policy makers. Findings of this study suggest that there is an urgent need for updating the EMLc in India. Regular updation of the EMLc can be helpful for regular access of necessary medicines which can reduce pediatric morbidity and mortality by providing quality healthcare at an affordable price.
